# Mixed Psyllium Fiber Improves the Quality, Nutritional Value, Polyphenols and Antioxidant Activity of Rye Bread

**DOI:** 10.3390/foods12193534

**Published:** 2023-09-22

**Authors:** Agata Wojciechowicz-Budzisz, Ewa Pejcz, Radosław Spychaj, Joanna Harasym

**Affiliations:** 1 Department of Biotechnology and Food Analysis, Wroclaw University of Economics and Business, Komandorska 118/120 Street, 53-345 Wrocław, Poland; ewa.pejcz@ue.wroc.pl (E.P.); joanna.harasym@ue.wroc.pl (J.H.); 2 Department of Fermentation and Cereals Technology, Wrocław University of Environmental and Life Sciences, J. Chełmońskiego 37 Street, 51-630 Wrocław, Poland; radoslaw.spychaj@upwr.edu.pl

**Keywords:** *Plantago*, psyllium, rye bread, dietary fiber, antioxidant activity

## Abstract

The aim of the study was to determine the influence of the different shares (0/100, 5/95, 10/90 and 15/85 ratios) of a ground psyllium fiber (PF) mixture of 80% psyllium seeds (*Plantago psyllium*) and 20% psyllium husk (*Plantago ovata Forsk*) on the quality characteristics, chemical composition, total polyphenolic content (TPC), and antioxidant activity of rye bread (RB). The study was conducted with rye flour (RF) type 580 and 720 and two dough preparation methods (single-phase—1F, two-phase—2F). The inclusion of psyllium fiber in rye bread resulted in an increase in the overbaking of bread by 12.4%, total protein by 1.7%, ash by almost twofold, and TDF content by more than twofold. Psyllium fiber addition also led to a twofold improvement in antioxidant activity and an increase in TPC from 35.5 to 109.1 mg GAE/100 g d.m., as well as enhanced porosity of the crumb from 7.1 to 7.6 points on the Mohs scale. However, it caused a decrease in specific loaf volume by 10%, springiness by 3.5%, chewiness by almost 12%, and gumminess of the crumb by 8.1%. A darkening of the crust (reduction in the L* value by 10.7%) and crumb (reduction in the L* value by 37.6%) was observed as well. Notably, the results indicated that a 10% share of PF can be considered a potentially beneficial and functional ingredient, promoting health benefits without negatively affecting the physical and sensory qualities of rye bread. This suggests the potential use of PF for enhancing the nutritional value of RB without compromising its overall quality.

## 1. Introduction

The development of numerous civilization diseases as a result of modern consumption patterns has become a concern. Overweight and obesity, affecting more than half of European populations, are among the most prevalent diet-related conditions [1]. The consumption of highly processed, low-fiber foods that are calorie-dense and nutritionally poor contributes to these issues. As a result, there has been a significant increase in consumer interest in functional foods, which have health-promoting effects on the human body. These products are rich in essential nutrients, minerals, vitamins, enzymes, and other bioactive ingredients, offering therapeutic benefits for specific diseases [1].

According to the World Health Organization (WHO), adults should consume 20–40 g of dietary fiber per day, but actual intake averages around 15 g. To address this deficiency, a commercial psyllium fiber (PF) was developed to enrich products with dietary fiber. PF is a combination of 80% psyllium seeds (*Plantago psyllium*) and 20% psyllium husk (*Plantago ovata Forsk*). Extensive research on the composition and effects of the different proportions of these ingredients revealed that a 4:1 ratio of soluble to insoluble fractions provided the best results in terms of satiety and overall composition [2].

Psyllium is a collective term used to describe the husk, seeds, and whole plant of the *Plantago* genus. Psyllium seeds contain high levels of fiber, protein, sterols, and aucubin. They stimulate the digestive tract, improve intestinal peristalsis, and are beneficial for digestive problems and metabolism. Psyllium seeds also lower levels of LDL (bad) cholesterol while increasing levels of HDL (good) cholesterol, making them especially valuable for individuals with heart disease, atherosclerosis, or other cardiovascular conditions [2,3]. Additionally, the *Plantago ovata* husk is rich in acidic mucus compounds and water-soluble fiber, which exhibit strong laxative effects. The husk’s fiber absorbs water and toxins, facilitating the removal of intestinal deposits from the body. Both the husk and seeds of *Plantago ovata* support intestinal health, improving peristalsis, providing a laxative effect, and countering diarrhea [3]. Psyllium fiber supplementation may also aid in weight loss [4]. Soluble fiber products like psyllium, when added to a diet, improve blood lipid profiles and the glycemic response, and they increase satiety [5]. By combining the benefits of both *Plantago psyllium* whole seeds and *Plantago ovata* husk, a psyllium fiber mixture can provide a more holistic approach to fiber supplementation, addressing different digestive concerns and offering a comprehensive range of benefits for digestive health.

Rye bread is traditionally made using a sourdough fermentation process involving lactic acid fermentation with lactic bacteria and alcohol fermentation with yeast. This method results in a low glycemic index, high prebiotic content, and a reduction in anti-nutritional ingredients. Sourdough bread is also characterized by a unique aroma derived from the complex process of flour acidification, fermentation, and baking [6]. Traditionally, spontaneous fermentation is carried out in rye dough using the microorganisms naturally present in the flour. In recent years, in order to accelerate the production of rye bread, direct the fermentation process, as well as improve the quality and safety of the finished product, the addition of starter cultures with or without yeast has been used.

Although many studies have examined the health effects of psyllium [3,7,8], there is a scarcity of research on the development of psyllium-enriched food products, particularly rye bread. While studies have explored gluten-free [9,10,11,12,13] and wheat bread [14,15,16], the impact of psyllium on rye bread quality remains largely unexplored. Therefore, there is a growing need to promote the health benefits of supplementation and encourage the utilization of psyllium fiber in food products. The aim of the conducted research was to compare the effect of different amounts of psyllium fiber (PF) (mixture of psyllium seeds—80% and psyllium husk—20%) on the quality, nutritional value, and polyphenolic and antioxidant activity of rye bread (RB) baked from two types of rye flour (RF) using the single-phase (1F) and two-phase method (2F).

## 2. Materials and Methods

### 2.1. Materials

The study was conducted with RF type 580 (moisture 10.5%, total protein 6.7%, ash 0.52 g/100 g) and type 720 (moisture 9.7%, total protein 12.0%, ash 0.69 g/100 g) (GoodMills Polska Ltd., Stradunia, Poland) and psyllium fiber (PF) (80% psyllium seeds (*Plantago psyllium*) and 20% psyllium husk (*Plantago ovata Forsk*) (moisture 6.8%, total protein 17.7%, ash 2.8 g/100 g) (Agnex, Białystok, Poland)). PF was grinded using a KT 120 laboratory hammer type mill. Compressed yeast and commercial freeze-dried Saf Levain LV2 starter cultures (*Saccharomyces chevalieri*, *Lactobacillus casei,* and *Lactobacillus brevis*) were supplied by Lesaffre Bio-Corporation Inc. (Warsav, Poland). Light rye sourdough in paste form was supplied by the Uldo Polska company (Wrocław, Poland). Other ingredients such as salt were purchased from a local store.

#### Dough Formulations and Bread Baking

Rye bread (RB) was baked with the single-stage (1F) and two-phase (2F) method. In both methods, PF was used to prepare blends with RF in 0/100, 5/95, 10/90, and 15/85 ratios. The control sample was 100% RB.

In the direct method (1F), the dough was prepared using 250 g of RF or an RF/PF blend; yeasts—1.0 g/100 g of flour, salt—1.5 g/100 g of flour, light rye sourdough in paste form —5.0 g/100 g of flour were combined in a Sigma mixer S 300 (Brabender farinograph, Duisburg, Germany) at 63 rpm by adding water at a temperature of 30 °C, until the consistency of the dough was 250 ± 20 FU. The obtained dough was placed in metal baking pans (8.5 × 8.5 cm at the base, 13 × 13 cm at the top edge, 10.5 cm in height) greased with oil. The pans were placed in a fermentation chamber (IBIS, Szubin, Poland), where the dough was allowed to ferment for 90 min (at a temperature of 30 °C, relative humidity of 85%). Afterward, the dough was manually degassed and left for final fermentation (rye dough from RF type 580—72–80 min, rye dough from RF type 720—60–67 min).

In the two-phase (2F) method, acid was first prepared by fermenting a mixture of RF (50% from the recipe) with water and the addition of 0.5% LV2 starter cultures for 18 h at 28–29 °C at a yield of 180 phase. The dough was prepared using previously made sourdough with pH = 4.0–4.2 (containing 50% of flour from the recipe), 50% of flour from a recipe for 250 g of flour or a RF-F blend, yeasts—1.0 g/100 g of flour, salt—1.5 g/100 g of flour, and the amount of water at a temperature of 30 °C needed to obtain dough with a consistency of 250 ± 20 FU. Fermentation was carried out at the temperature of 30 °C until oven maturity was obtained (rye dough from RF type 580—94–101 min, rye dough from flour type 720—107–123 min).

The bread was baked in two replications in a GT 800 electric furnace (IBIS, Szubin, Poland), at a temperature of 250 °C for 30 min, with steaming in the first 3 min of baking. After baking, the loaves were sprinkled with water and left at room temperature to cool down. Each sample of bread after 24 h was freeze-dried (Labconco Corporation, Kansas City, MO, USA) and milled (laboratory mill WŻ1, Sadkiewicz Instruments, Bydgoszcz, Poland).

### 2.2. Methods

#### 2.2.1. Bread Characteristics

After 24 h, the bread was evaluated in terms of specific volume, porosity of the crumb according to the Mohs scale, crust and crumb color, and texture profile analysis (TPA). After cooling, the bread was weighed to determine overbaking with respect to the weight of the flour used for baking. Bread volume was assessed by the millet displacement method using the SA-WY bread volumeter (ZBPP, Bydgoszcz, Poland) filled with millet grain and expressed in cm^3^ per 1 g of bread [11]. The porosity of the crumb was assessed according to a visual 10-point Mohs scale, where 1 point was awarded to the sample with large and uneven pores, and 10 points—to the crumb with fine, even porosity with thin walls and uniform thickness. Analyses were performed at least in duplicate. Bread crust and crumb color was measured with a Minolta Colorimeter (CR-400/410, Konica Minolta, Japan). Five different points of the slice (crumb) and loaf surface (crust) with L*, a*, and b* values were measured. 

The texture of the crumb was determined 24 h after baking. The properties of the bread crumb were determined using the TPA method performed on a ZWICK ROEL Z010 apparatus with a 100 N load cell (Zwick Roell, Ulm, Germany). A sample of the crumb in the form of a cylinder with height of h = 13 mm and a diameter of Ø = 17 mm was subjected to double compression (40% of compression) by a flat plate (Ø = 35 mm) with a falling speed of 60 mm/min with an interval of 0.5 min between compression cycles. Based on the collected data, the following parameters were obtained: hardness [N]; springiness; cohesiveness; chewiness [N*mm]; and gumminess [N]. All samples were tested in 8 replicates. 

#### 2.2.2. The Chemical Composition of the Bread

The bread samples were determined for the following: moisture—with the AACC Approved Method 44–15.02 [17], total protein content—with the Kjeldahl method using a Foss Tecator Kjeltec 2400 analyzer (Foss, Hilleroed, Denmark) (N×5.7), ash content—with the AACC Method 46.11A, and total dietary fiber content (TDF)—acc. to AOAC Method 985.29 [18] using total dietary fiber assay kits TDF-100A-1KT and TDF-C10 (Sigma-Aldrich, Saint Louis, MI, USA). The samples were analyzed at least in duplicate, and the results were expressed on a dry matter (d.m.) basis.

#### 2.2.3. The Determination of Total Polyphenolic Content (TPC) and Antioxidant Activity

The total polyphenolic content (TPC) of the bread samples was determined using the Folin–Ciocalteu spectrophotometric method [19]. Absorbance at 765 nm was measured after 1 h using the UV-2401 PC spectrophotometer (Shimadzu, Kyoto, Japan). Results were expressed as mg of gallic acid equivalents (GAE) per 100 g of dry bread (mg GAE/100 g d.m.). The ABTS and FRAP methods were applied in our studies as reported by Re et al. [20] and Benzie et al. [21]. Absorbance was measured at 734 nm and 593 nm using the UV-2401 PC spectrophotometer (Shimadzu, Kyoto, Japan). The results of the antiradical capacity and reducing power were expressed as Trolox equivalents in μmol per 100 g of dry sample (μM TE/100 g d.m.). Data were expressed as the mean value for three measurements.

### 2.3. Statistic Analysis

The results presented are mean values ± standard deviation (SD). Statistical analysis such as one-way and three-way ANOVA were analyzed using Statistica 13.3 (StatSoft, Kraków, Poland). Significant differences (*p* ≤ 0.05) between the mean values were determined using Duncan’s Multiple Range Test. Principal Component Analysis (PCA) was performed to determine correlations between the quality traits, texture, nutritional content, antioxidant activity, and TPC of RB enriched with PF (Statistica 13.3).

## 3. Results and Discussion

### 3.1. The Quality Traits and Nutritional Content of the Bread

The quality features of the bread were directly influenced by the raw materials used, including additional materials, production parameters, and the way of carrying out the dough. The quality traits and nutritional content of RB enriched with PF are presented in Table 1. With the increase in the amount of PF in the samples, the specific volume of bread decreased. The highest value was found in the control bread sample (100% RF) and with a 5% share of PF baked from RF type 720 using the 1F method. The lowest value of specific volume was characteristic for the bread samples with 10% and 15% PF content from RF type 720, carried out by the 2F method. Loaf specific volume (cm^3^/g) gave us information about the volume-to-weight ratio. Dough with PF was characterized by higher water absorption than RF dough, which resulted in a higher weight of dough and bread and, at the same time, a lower specific volume. In wheat dough containing raw materials with a high dietary fiber content , the protein system was weakened and had a lower ability to retain gases. A decrease in specific volume with psyllium supplementation was also observed by others [11,12,15,16,22,23]. On the other hand, Pejcz and Burešová [11] and Aprodu and Banu [24] noted that *Plantago* additives resulted in an increase in the volume of bread obtained from 100 g of flour. The increase in specific loaf volume of gluten-free bread as a result of the addition of psyllium husk powder was observed by Fratelli et al. [13]. These authors explained that increased bread volume could be related to psyllium hydrocolloids’ gelling ability, which strengthens gas cells and helps their expansion. Ziemichód et al. [25] stated that bread baked with a share of *Plantago psyllium* in the form of whole and ground seeds were characterized by a larger bread volume than the control bread, but the bread with a share of whole *Plantago ovata* seeds did not differ significantly from the bread without the additive.

However, in the case of ground *Plantago ovata* seeds, the specific volume of bread was the lowest. This result was affected by the value of the bread weight, which was observed to be the highest for bread with a share of *Plantago ovata* seeds; the dough absorbed the most water. Bread obtained by the 1F method from both types of RF were characterized by an increasing overbaking capacity along with an increasing share of PF in the samples, where the highest value was obtained for the bread with a 15% share of PF (81.6 ± 1.8% and 83.8 ± 1.0%). When using the 2F baking method for both types of RF, the control bread (0%) and bread with 5% PF content had a lower overbaking capacity than the samples with 10 and 15% PF content. The overbaking increase as a result of *Plantago* product incorporation could reflect their high water absorption and water retention capacity during baking [11,16]. Many authors found such dependence to be due to the addition of fiber preparations to cereal products [23].

The crumb porosity of bread baked using the 2F method from RF type 720 was rated the highest (9 ± 0.0 points), while the lowest was rated from bread baked with 5% PF from type 580 flour, both methods, and 720 with the 1F method (6 points). With lower scores, the pores were large and uneven, and with higher scores, they were fine and even, as shown in Figure S1 (Supplementary Data).

The lowest content of total protein, ash, and TDF was found in control bread baked with RF type 580, obtained using two methods. With the increase in the share of PF in the samples, these values increased, reaching the highest value with a 15% share of PF in bread made from RF type 720 using the 1F method. Similar changes in the content of dietary fiber and protein in bread with the addition of *Plantago* seeds and husk were described by Pejcz et al. [26]. In the study of Beikzadeh et al. [27] increasing husk percentages in sponge cakes caused a significant increase in ash and fiber content and also in the protein content. Aldughpassi et al. [28] stated that the TDF content in Arabic flatbread containing 3% and 5% psyllium husk increased significantly. Fratelli et al. [13] also reported increased fiber content in gluten-free bread with psyllium husk powder addition.

By analyzing the results from the 3-factor ANOVA, it was found that the type of RF had no effect on specific volume and overbaking (Table 1), while for the porosity of the crumb, total protein, ash, and TDF, higher values were recorded for bread baked with RF type 720 than 580. Using the 1F baking method, higher values for specific volume, overbake, total protein, and TDF were obtained than with the 2F method. However, the porosity of the 2F bread samples was rated higher than for the 1F bread samples. Along with the increasing share of PF, the values of all the features increased. Only in the case of specific volume it was different, where higher values were recorded for the control bread and with a 5% share of PF than with the 10 and 15% share of PF. In the case of specific volume, the interaction of the baking method and RF type, as well as baking method and PF share, had a significant impact. In the case of crumb porosity and ash, there was an interaction between the baking method and type of RF. When analyzing the influence of factors on total protein content, a significant interaction effect of the baking method and RF type, as well as RF type and PF content, was found.

### 3.2. Total Polyphenolic Content (TPC) and Antioxidant Activity

The TPC and the antioxidant activity of the bread samples are presented in Table 2. As expected, the enrichment of the bread with PF showed a positive effect on TPC, the antiradical activity (ABTS assay), and reducing power (FRAP assay). Pejcz et al. [26] made similar observations in their research. The highest TPC, antiradical activity, and reducing power was characterized by bread made of 720 type RF, baked with 2F method, with 15% PF share and the lowest by control bread made of 580 type RF, baked with 2F method. It is well known that the antioxidant capacity of food products is strongly correlated with polyphenol content [29]. Control bread made of RF type 580 and baked by the 1F method characterized the lowest ABTS and FRAP. The control bread made of 720 type RF and baked with the 1F method also had the lowest antioxidant activity against the ABTS radical.

By analyzing the results from the 3-factor ANOVA, it was found that the 720 type RF bread had a significantly higher TPC, antioxidant capacity, and reducing power than RF type 580. The dough preparation method had no effect on ABTS. In the case of TPC and FRAP, a higher value was obtained for the 2F method. In the study of Konopka et al. [30], the high polyphenol content of rye flour decreased significantly during the bread making process (fermentation and baking), while the total antioxidant activity (both ABTS and FRAP) increased significantly during baking. Pejcz et al. [31] observed that bread produced by the single-phase method had higher polyphenolic content and ABTS antioxidant activity, possibly due to the higher acidity and composition of the baking acid paste. Michalska et al. [32] explained that the baking process influences the overall antioxidant activity of rye bread by the formation of Maillard compounds during baking. In our study, with the increasing amount of PF in the bread, the antioxidant activity and the TPC increased. In the case of ABTS, the interaction of the baking method and the type of RF had a significant impact. In the case of FRAP, all double interactions (baking method and RF type, baking method and PF share, RF type and PF share) and the triple interaction had a significant impact. However, in the case of TPC, the interaction of the baking method and type of RF, baking method and PF share, and the triple interaction had a significant impact.

### 3.3. The Color of the Crust and Crumb of the Bread

The color parameters of the crust and crumb of RB enriched with PF are presented in Table 3. According to the research conducted by Pejcz et al. [26], the color of the bread could be influenced by the polyphenols contained in *Plantago* seeds and psyllium husk. One of the main causes of the color change is the natural pigments present in the psyllium seed husk. The enrichment of RF with PF resulted in the decreased values of almost all the assessed attributes. Bread with RF type 580, 2F, and 5% PF was characterized by the lightest color and the highest share of yellow in the color of the crust, while the darkest color of the crust was that of bread with RF type 720, 1F, 10% PF. The control bread with RF type 580, both 1F and 2F, had the highest share of red in the color of the crust, while the lowest share of red and yellow in the crust color was in bread with RF type 720, 1F, 15% PF. The lightest crumb was characteristic for the control bread with RF type 580, 2F; the darkest crumb was characteristic for the bread with RF type 720, 1F, 15% PF. The reddest was the bread crumb of bread with RF type 580, 2F, 15% PF; the greenest was the bread crumb of bread with RF type 580, 2F, 0% PF. The most yellow was the crumb of all the control loaves, and the least yellow was the crumb of the bread with RF type 720, 1F, 15% PF.

As a result of the 3-factor ANOVA analysis, it was found that a lighter color of the crust and crumb and a higher proportion of red and yellow in the color of the crust was characteristic of bread baked with 580 type RF than with 720 type RF. A lighter color of the crust and crumb, a higher proportion of red in the color of the crust and yellow in the color of the crumb, and a lower proportion of red in the color of the crumb were characteristic of the bread obtained using the 2F method rather than the 1F method. With the increase in the share of PF in the bread, the color of the crust and crumb darkened; the share of red and yellow in the color of the crust and yellow in the color of the crumb decreased. The share of red in the crumb color for the control bread was lower than for PF-enriched bread. In the research of Pejcz et al. [26] *Plantago* additives did not affect the brightness of the crust, while the increasing share of these additives contributed to the decrease in crumb brightness. These results agree with those of Ziemichód et al. [25]. Ziemichód et al. [25] and Pejcz et al. [26] made the same observations, like ours, regarding the influence of the used additives on crumb a* and b* color factors. Similar observations of the change in crumb color caused by the addition of psyllium husk to biscuits were made by Beikzadeh et al. [27]. Aldughpassi et al. [28] stated that the addition of psyllium husk to wheat pita bread decreased the lightness and the b* value (yellowish color), and there was no change in the red color of the crumb. In the case of the a* crust color discriminant, the interaction of the type of RF and the PF share as well as the triple interaction had a significant impact. In the case of the b* crust color discriminant, the interaction of the RF type and the PF share also had a significant impact. In the case of the L* crumb color discriminant, the interaction of the baking method and PF share, RF type and PF share, and the triple interaction had a significant impact. In the case of the a* crumb color discriminant, all interactions had a significant effect, and in the case of b*, the baking method and RF type, as well as the triple interaction, had a significant impact.

### 3.4. Texture Profile Analysis (TPA)

The enrichment of RB with PF influenced TPA parameters (Table 4). Bread with RF type 580, baked with the methods 1F and 2F with the participation of PF, were characterized by lower hardness than the control sample. With the increase in the amount of PF in the RF type 720, 1F bread, crumb hardness gradually increased (from 3.55 ± 0.63 to 6.87 ± 0.59 N). An increase in the hardness of biscuits under the influence of 15% psyllium husk incorporation was observed by Beikzadeh et al. [27].

The lowest crumb hardness was found in the control bread made of 720 type RF, 1F method; the hardest were bread baked with 720 type RF, carried out by the 2F method with a PF share of 0–10%. In the studies by Pejcz & Burešová [11] and Ziemichód et al. [25], the hardness of the fresh gluten-free bread with *Plantago* additives was lower than that of the control bread. 

These results agree with those of Santos et al. [33], who studied the impact of psyllium addition on gluten-free bread. Abdullah et al. [34] stated that the addition of psyllium husk made the wheat bread texture significantly softer with increasing levels. Fratelli et al. [13] stated that psyllium husk powder addition decreased the crumb firmness of gluten-free bread. Cappa et al. [35] found that psyllium supplementation has to be carefully modulated, as it can determine an excessive increase in bread hardness if a proper amount of water is not added to the dough.

Bread cohesiveness is the ability to withstand compressive or tensile stress. The highest value of cohesiveness was obtained for the RF type 580, 1F, 10% PF bread and the lowest for the RF type 720, 2F, 10% PF bread. Bread baked from RF type 580 using the 1F method enriched with PF had a higher cohesiveness value than the control sample. The situation is the opposite in the case of bread baked from RF type 720 by the 1F method; the cohesiveness gradually decreases along with the increase in the share of PF in the bread from 0.65 ± 0.04 to 0.54 ± 0.04. Ziemichód et al. [25] noticed that bread with the seeds of *Plantago psyllium* or *Plantago ovata* in ground form had more than twice the cohesiveness of the control bread.

Regarding springiness, bread baked using the 1F method from both types of RF showed a visible decrease in the tested characteristic only containing a 15% share of PF. The RF type 580, 2F control bread had the highest springiness; the addition of PF caused its reduction. Bread with 0% and 5% PF from the 720 type RF, 2F method was characterized by higher springiness than those with 10 and 15% PF, which obtained the lowest values of the tested characteristic. Contrary conclusions regarding the springiness of gluten-free bread with the use of *Plantago* additives were drawn by Pejcz & Burešová [11].

The highest gumminess was for the RF type 720, 2F control bread; the lowest gumminess and chewiness was for the RF type 720, 1F control bread. In the case of type 580 flour loaves, the addition of PF caused a reduction in gumminess and chewiness in both baking methods. For bread made of 720 type RF and using the 1F method, the gumminess and chewiness of the crumb of the bread with PF increased, while the value of the tested characteristics decreased with the use of the 2F method. The highest value of chewiness was obtained for the RF type 580, 2F control bread. In the study of Pejcz & Burešová [11] bread chewiness was decreased by the incorporation of *Plantago* products.

As a result of the 3-factor ANOVA analysis, it was found that the type of RF had no effect on the gumminess. Bread baked with 580 type RF were characterized by higher values of such parameters as cohesiveness, springiness, chewiness, and lower hardness than those baked with RF type 720. Bread baked using the 1F method were characterized by higher cohesiveness and springiness and lower hardness, gumminess, and chewiness than those baked using the 2F method. The growing share of PF in bread did not affect hardness and cohesiveness but caused a decrease in springiness and chewiness. In the case of gumminess, the highest value was obtained for bread with a 5% share of PF and the lowest—15%. In the case of hardness, all double interactions had a significant effect. In the case of cohesiveness, however, all interactions had a significant impact. Analyzing the impact of the interaction on springiness, it was found that the interaction of the baking method and RF type had a significant impact; regarding gumminess, the interaction of baking method and RF type, baking method and PF share, and triple interaction had a significant impact. And regarding chewiness, the interaction of baking method and PF share and triple interaction had a significant impact.

### 3.5. Principal Component Analysis (PCA)

**Figure 1:**
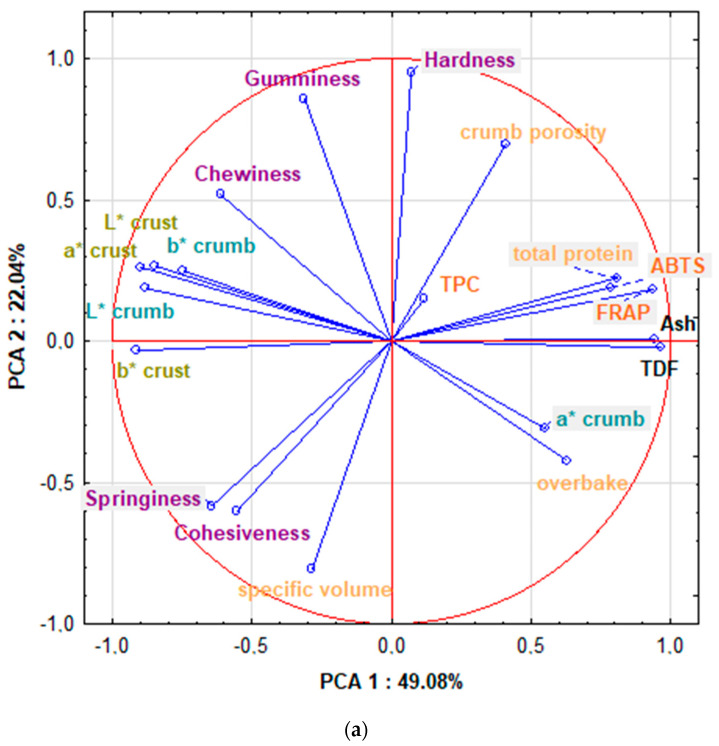

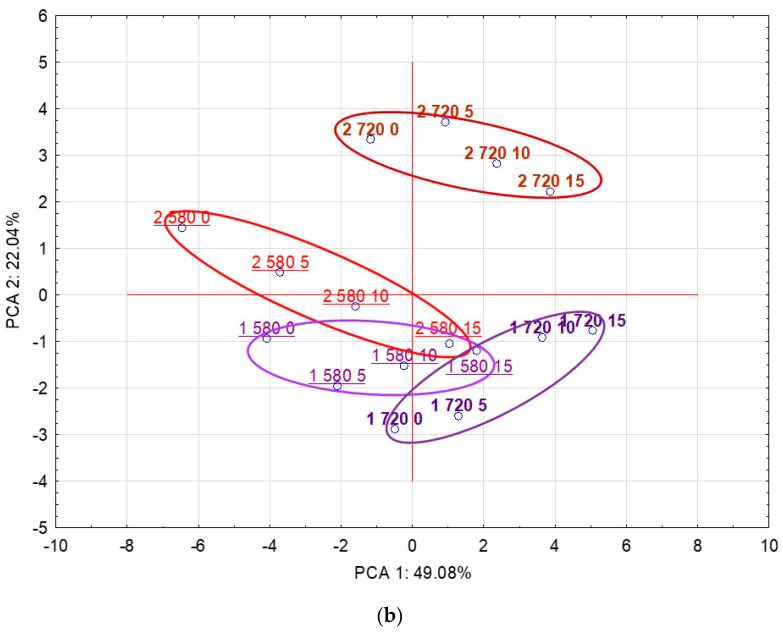
Principal component analysis diagram (**a**) PCA of quality traits, texture, nutritional content, and antioxidant activity of bread, TDF—total dietary fiber, TPC—total polyphenolic content; (**b**) PCA of differences and similarities between bread prepared with the 1F and 2F method with RF type 580 and 720 and with a PF share from 0 to 15%, 1 580 0—bread prepared by the 1F method, with RF type 580 without PF, 2 580 5—bread prepared by the 2F method, with RF type 580 with 5% PF, 1 720 10—bread prepared by the 1F method, with RF type 720 with 10% PF, 2 720 15—bread prepared by the 2F method, with RF type 720 with 15% PF.

The PCA allowed for the determining of correlations between the quality traits, texture, nutritional content, antioxidant activity, and TPC of RB enriched with PF (Figure 1a,b). The significant values of the Pearson’s correlation coefficients determined in the study are included in the Supplementary Data, Table S1.

The sum of the principal components determined for the quality traits, texture, nutritional content, and antioxidant activity of RB with PF was 71.12% (PC1: 49.08%, PC2: 22.04%) (Figure 1a). It was found that the total protein content, ash, and TDF strongly (>0.822) correlated with the antioxidant properties of RB (Figure 1a, Table S1). This can be explained by the fact that wholegrain psyllium flour—rich in protein, fiber and ash—contains many antioxidants, including vitamins, flavonoids, trace elements, as well as phenolic acids, lignans, and phytoestrogens [2]. Benitez et al. [36] also found that wholegrain flour showed the highest ferric reducing antioxidant power. There was also a negative correlation between the content of the ingredients in bread above and its springiness (−0.874, −0.505,−0.546). The dietary fiber present in *Plantago* has a high ability to absorb water and creates a denser consistency of bread, which leads to less springiness in the crumb. Santos et al. [33] observed significant correlations between the increase in crumb hardness with the reduction in texture acceptance. In our research, such correlations were not noticeable. The total protein content negatively correlated with cohesiveness and chewiness (−0.837; −0.595). Quality features of the bread, such as specific volume and crumb porosity, did not correlate with the content of any of the determined ingredients of the bread (total protein, ash, TDF, TPC). However, a positive correlation was found between ash and TDF with overbaking (0.535, 0.574), which results from the increased water absorption of mixtures with a high ash and fiber content, which causes an increase in the mass of bread and overbaking. Santos et al. [22] reported a negative correlation of loaf-specific volume and crumb firmness in gluten-free bread with psyllium husk. A high level of psyllium increases dough consistency, limiting expansion and consequently resulting in a denser structure with a low specific loaf volume and high crumb firmness [26]. A similar relationship was also found in our research. 

The PCA showed the diversity of bread obtained with different methods depending on the RF used (Figure 1b) and divided the bread samples assessed in the study into four groups; the most different from the others was the bread obtained by the two-phase method from flour type 720 (2 720). Bread obtained with the first method from both types of flour (1 580 and 1 720) and method 2 from flour type 580 (2 580) with different amounts of the additive was largely similar.

In all groups, the amount of PF additive in ranges from 0 to 15% caused the samples to be arranged in the same way (from left to right along PCA 1), which resulted in a similar effect of the additive on the assessed bread.

## 4. Conclusions

Psyllium fiber is an underrated structure creating ingredient with numerous health promoting features. Its widespread usage via incorporation into bread matrices is one of the best strategies to promote higher fiber consumption. Based on the results obtained, it can be concluded that bread baked from rye flour, type 780, and using the two-phase dough preparation method were of higher quality and health-promoting value. Notably, the results indicated that a 10% share of PF can be considered a recommended share of this functional ingredient, promoting health benefits without negatively affecting the physical and sensory qualities of rye bread. This successful enrichment strategy can be widened to other matrices that are both wheat based or gluten-free. The additional bioactivity of psyllium fiber, which results in the increase in antioxidant activity, will improve not only the structure but also the nutritional value.

## Figures and Tables

**Table 1:** The quality traits and nutritional content of rye bread enriched with PF.

Rye Flour Type	Baking Method	Share of PF [%]	Specific Volume [cm^3^/g]	Over-Bake [%]	Porosity of the Crumb	Total Protein [g/100 g d.m.]	Ash [g/100 g d.m.]	TDF [g/100 g d.m.]
580	1F	0	2.58 ± 0.02 bc	66.2 ± 1.5 abc	6.5 ± 0.7 cd	5.36 ± 0.18 i	0.57 ± 0.00 j	7.02 ± 0.09 h
		5	2.72 ± 0.17 b	70.0 ± 1.5 abc	6.0 ± 0.0 d	6.12 ± 0.04 h	0.63 ± 0.05 i	10.04 ± 0.42 g
		10	2.60 ± 0.11 bc	75.8 ± 1.8 ab	8.0 ± 0.0 abc	6.81 ± 0.01 g	0.85 ± 0.05 fg	13.76 ± 1.12 ef
		15	2.43 ± 0.01 cd	81.6 ± 1.8 a	8.5 ± 0.7 ab	7.52 ± 0.17 f	0.96 ± 0.01 cd	17.00 ± 1.24 c
	2F	0	2.66 ± 0.16 bc	52.1 ± 1.4 c	6.5 ± 0.7 cd	5.48 ± 0.01 i	0.56 ± 0.01 j	6.69 ± 0.34 h
		5	2.56 ± 0.10 bc	51.5 ± 1.4 c	6.0 ± 0.0 d	6.12 ± 0.02 h	0.73 ± 0.01 h	9.74 ± 0.56 g
		10	2.50 ± 0.16 bc	57.7 ± 1.4 bc	7.0 ± 0.0 bcd	6.76 ± 0.01 g	0.81 ± 0.00 g	13.06 ± 0.54 ef
		15	2.63 ± 0.21 bc	57.1 ± 1.5 bc	6.5 ± 0.7 cd	7.47 ± 0.06 f	0.97 ± 0.01 cd	16.39 ± 1.10 cd
720	1F	0	3.06 ± 0.09 a	62.7 ± 1.5 abc	6.5 ± 0.7 cd	10.68 ± 0.13 d	0.74 ± 0.00 h	12.42 ± 1.07 f
		5	2.98 ± 0.04 a	67.7 ± 1.4 abc	6.0 ± 1.4 d	11.13 ± 0.11 c	0.90 ± 0.04 ef	16.08 ± 0.92 cd
		10	2.73 ± 0.01 b	73.1 ± 1.4 abc	7.5 ± 0.7 bcd	11.60 ± 0.01 b	1.00 ± 0.05 c	19.42 ± 0.18 b
		15	2.46 ± 0.06 bcd	83.8 ± 1.0 a	6.5 ± 2.1 cd	11.98 ± 0.09 a	1.15 ± 0.02 a	21.62 ± 1.10 a
	2F	0	2.40 ± 0.03 cde	57.6 ± 1.8 bc	9.0 ± 0.0 a	10.36 ± 0.02 e	0.72 ± 0.02 h	9.44 ± 0.99 g
		5	2.22 ± 0.01 de	59.2 ± 1.9 bc	9.0 ± 0.0 a	10.74 ± 0.01 d	0.85 ± 0.01 fg	14.64 ± 0.52 de
		10	2.15 ± 0.18 e	66.1 ± 1.7 abc	9.0 ± 0.0 a	11.22 ± 0.13 c	0.91 ± 0.00 de	17.42 ± 1.02 c
		15	2.15 ± 0.13 e	64.6 ± 1.8 abc	9.0 ± 0.0 a	11.67 ± 0.03 b	1.07 ± 0.01 b	20.92 ± 0.18 ab
580			2.59 ± 0.13 a	63.9 ± 1.9 a	6.9 ± 0.9 b	6.45 ± 0.80 b	0.76 ± 0.16 b	11.71 ± 1.87 b
720			2.52 ± 0.36 a	66.8 ± 1.3 a	7.8 ± 1.4 a	11.17 ± 0.54 a	0.92 ± 0.15 a	16.50 ± 1.16 a
1F			2.70 ± 0.23 a	72.6 ± 1.6 a	6.9 ± 1.2 b	8.90 ± 2.62 a	0.85 ± 0.19 a	14.67 ± 1.72 a
2F			2.41 ± 0.23 b	58.1 ± 1.8 b	7.8 ± 1.3 a	8.72 ± 2.43 b	0.83 ± 0.16 a	13.54 ± 1.63 b
0			2.68 ± 0.27 a	59.4 ± 1.7 b	7.1 ± 1.2 ab	7.97 ± 2.73 d	0.65 ± 0.09 d	8.89 ± 1.60 d
5			2.62 ± 0.30 a	62.1 ± 1.6 ab	6.8 ± 1.5 b	8.53 ± 2.58 c	0.78 ± 0.12 c	12.62 ± 1.69 c
10			2.50 ± 0.25 b	68.2 ± 1.3 ab	7.9 ± 0.8a	9.09 ± 2.48 b	0.89 ± 0.12 b	15.92 ± 1.01 b
15			2.42 ± 0.20 b	71.8 ± 1.04 a	7.6 ± 1.5 a	9.66 ± 2.32 a	1.04 ± 0.10 a	18.98 ± 1.58 a

Values are expressed as the mean (n = 2) ± standard deviation. Mean values bearing different letters in the same row denote statistical differences (a > b > c …, etc.). PF—psyllium fiber, 1F—one-phase baking method, 2F—two-phase baking method.

**Table 2:** The Total Polyphenolic Content (TPC) and Antioxidant Activity of rye bread enriched with PF.

Rye Flour Type	Baking Method	Share of PF [%]	TPC [mg GAE/100 g d.m.]	ABTS [µM TE/100 g d.m.]	FRAP [µM TE/100 g d.m.]
580	1F	0	19.3 ± 0.56 ij	157.1 ± 1.1 i	213 ± 1.1 k
		5	46.9 ± 0.99 h	222.3 ± 1.2 ef	337.0 ± 1.3 j
		10	62.3 ± 1.96 fg	227.1 ± 1.0 e	624.8 ± 3.1 gh
		15	106.6 ± 1.12 de	315.7 ± 1.7 b	924.5 ± 4.1 c
	2F	0	2.8 ± 0.24 k	166.9 ± 0.7 i	255.0 ± 1.2 k
		5	14.4 ± 0.32 jk	201.2 ± 1.0 gh	509.1 ± 2.1 i
		10	31.7 ± 0.32 i	230.9 ± 1.1 e	874.8 ± 3.4 d
		15	48.0 ± 0.87 h	293.2 ± 1.0 cd	951.0 ± 5.2 c
720	1F	0	62.7 ± 1.39 fg	166.5 ± 0.4 i	585.2 ± 2.1 h
		5	73.2 ± 1.49 f	211.1 ± 0.8 fg	631.8 ± 2.7 g
		10	122.8 ± 1.24 bc	303.8 ± 1.2 bc	751.4 ± 3.4 e
		15	132.2 ± 1.95 b	286.7 ± 0.9 d	869.0 ± 4.1 d
	2F	0	57.3 ± 0.49 gh	197.2 ± 1.5 h	695.5 ± 2.3 f
		5	99.1 ± 1.56 e	305.8 ± 1.1 bc	929.9 ± 5.2 c
		10	116.9 ± 1.76 cd	234.6 ± 0.6 e	1310.9 ± 4.6 b
		15	149.6 ± 1.72 a	331.5 ± 1.1 a	1453.4 ± 5.2 a
580			41.5 ± 0.95 b	226.8 ± 1.1 b	586.1 ± 1.49 b
720			101.07 ± 0.92 a	254.6 ± 1.3 a	903.4 ± 1.54 a
1F			65.0 ± 1.12 b	236.3 ± 1.1 a	617.1 ± 1.38 b
2F			78.2 ± 1.38 a	245.1 ± 1.2 a	872.4 ± 1.66 a
0			35.5 ± 0.89 d	176.9 ± 1.7 c	437.2 ± 1.4 d
5			58.4 ± 0.90 c	239.1 ± 1.5 b	601.9 ± 1.39 c
10			83.4 ± 1.25 b	243.5 ± 1.1 b	890.5 ± 1.56 b
15			109.1 ± 1.31 a	311.6 ± 1.1 a	1049.5 ± 2.46 a

Values are expressed as the mean (n = 2) ± standard deviation. Mean values bearing different letters in the same row denote statistical differences (a > b > c …, etc.). PF—psyllium fiber, 1F—one-phase baking method, 2F—two-phase baking method.

**Table 3:** The color parameters of the crust and crumb of rye bread enriched with PF.

Rye Flour Type	Baking Method	Share of PF [%]	The Crust Color			The Crumb Color		
			L*	a*	b*	L*	a*	b*
580	1F	0	36.16 ± 1.11 abc	10.21 ± 0.39 a	15.54 ± 1.16 ab	50.69 ± 1.24 bc	2.82 ± 0.55 h	17.94 ± 1.44 a
		5	33.97 ± 1.40 bcd	8.25 ± 0.45 b	12.52 ± 0.93 bc	43.71 ± 0.20 d	4.85 ± 0.30 bcd	12.23 ± 1.85 bc
		10	32.54 ± 1.74 def	7.05 ± 0.52 bcd	9.85 ± 1.60 cd	40.61 ± 1.65 def	5.02 ± 0.30 bc	9.74 ± 1.35 bcde
		15	31.28 ± 0.20 defg	5.59 ± 0.23 ef	7.0 ± 0.90 de	36.96 ± 2.09 fgh	4.48 ± 0.19 de	7.08 ± 1.00 efg
	2F	0	38.78 ± 2.30 ab	10.60 ± 0.54 a	15.51 ± 0.81 ab	68.77 ± 0.72 a	-1.67 ± 0.24 i	19.72 ± 0.29 a
		5	40.0 ± 3.09 a	8.36 ± 0.90 b	16.48 ± 1.34 a	48.92 ± 1.21 bc	4.66 ± 0.05 cde	11.70 ± 0.15 bcd
		10	36.14 ± 0.41 abc	7.47 ± 0.10 bc	10.96 ± 0.39 c	41.73 ± 1.18 de	5.20 ± 0.12 b	8.88 ± 0.26 cdef
		15	31.8 ± 0.08 defg	5.06 ± 0.25 f	3.71 ± 0.67 efg	33.48 ± 1.06 hi	6.06 ± 0.06 a	4.08 ± 0.08 gh
720	1F	0	31.28 ± 2.64 defg	6.52 ± 0.34 cde	6.68 ± 1.37 def	48.19 ± 2.03 c	3.88 ± 0.10 g	17.13 ± 1.75 a
		5	30.02 ± 3.35 efg	5.11 ± 0.37 f	4.92 ± 0.56 efg	41.52 ± 2.03 de	4.71 ± 0.26 bcde	11.20 ± 1.17 bcd
		10	26.35 ± 2.77 g	3.84 ± 0.10 g	3.23 ± 1.07 fg	36.08 ± 2.21 gh	4.32 ± 0.13 efg	6.16 ± 1.17 fg
		15	27.84 ± 2.96 fg	2.31 ± 0.56 h	1.72 ± 0.21 g	31.14 ± 1.92 i	3.95 ± 0.08 fg	1.31 ± 0.11 h
	2F	0	31.93 ± 2.04 defg	7.43 ± 0.67 bc	5.61 ± 0.89 ef	52.54 ± 1.22 b	2.44 ± 0.24 h	19.98 ± 0.19 a
		5	34.09 ± 1.37 bcd	7.61 ± 0.33 bc	7.27 ± 1.26 de	42.0 ± 1.75 de	4.22 ± 0.16 efg	12.92 ± 0.27 b
		10	32.62 ± 0.01 def	6.73 ± 0.25 cde	5.52 ± 0.39 ef	38.0 ± 0.84 efg	4.48 ± 0.04 de	10.30 ± 0.33 bcde
		15	32.08 ± 0.30 defg	6.13 ± 0.43 ef	4.33 ± 0.95 efg	35.47 ± 0.11 gh	4.41 ± 0.03 def	8.70 ± 0.24 def
580			35.11 ± 1.30 a	7.82 ± 1.94 a	11.44 ± 1.45 a	45.61 ± 1.62 a	3.93 ± 0.36 a	11.42 ± 2.16 a
720			30.78 ± 1.33 b	5.71 ± 1.84 b	4.91 ± 1.92 b	40.62 ± 1.00 b	4.05 ± 0.69 a	10.96 ± 2.84 a
1F			31.21 ± 1.78 b	6.11 ± 1.44 b	7.68 ± 1.53 a	41.11 ± 1.47 b	4.26 ± 0.71 a	10.35 ± 1.59 b
2F			34.68 ± 1.36 a	7.42 ± 1.62 a	8.68 ± 1.21 a	45.11 ± 1.16 a	3.72 ± 0.33 b	12.04 ± 1.30 a
0			34.53 ± 1.66 a	8.69 ± 1.91 a	10.84 ± 1.40 a	55.05 ± 1.73 a	1.87 ± 0.27 b	18.69 ± 1.71 a
5			34.52 ± 1.24 a	7.33 ± 1.55 b	10.30 ± 1.89 a	44.04 ± 1.43 b	4.61 ± 0.30 a	12.01 ± 1.30 b
10			31.91 ± 1.41 ab	6.28 ± 1.54 c	7.39 ± 1.28 b	39.10 ± 1.78 c	4.72 ± 0.41 a	8.77 ± 1.14 c
15			30.81 ± 1.38 b	4.77 ± 1.60 d	4.19 ± 0.74 c	34.26 ± 1.73 d	4.76 ± 0.86 a	5.29 ± 1.47 d

Values are expressed as the mean (n = 2) ± standard deviation. Mean values bearing different letters in the same row denote statistical differences (a > b > c …, etc.). PF—psyllium fiber, 1F—one-phase baking method, 2F—two-phase baking method.

**Table 4:** The parameters of the Instrumental Test of Texture Profile Analysis (TPA) of rye bread enriched with psyllium fiber.

Rye Flour Type	Baking Method	Share of PF [%]	Hardness [N]	Cohesiveness	Springiness	Gumminess [N]	Chewiness [N*mm]
580	1F	0	5.62 ± 0.85 def	0.67 ± 0.02 de	0.92 ± 0.03 ab	3.75 ± 0.56 e	3.44 ± 0.48 de
		5	5.20 ± 0.84 ef	0.73 ± 0.02 ab	0.92 ± 0.02 ab	3.78 ± 0.54 e	3.48 ± 0.47 de
		10	5.04 ± 0.55 efg	0.75 ± 0.02 a	0.91 ± 0.02 ab	3.75 ± 0.45 e	3.42 ± 0.44 de
		15	4.94 ± 0.35 efg	0.73 ± 0.02 ab	0.90 ± 0.02 b	3.59 ± 0.43 ef	3.23 ± 0.42 e
	2F	0	7.86 ± 0.90 b	0.68 ± 0.02 cd	0.95 ± 0.01 a	5.36 ± 0.55 abc	5.09 ± 0.32 a
		5	7.63 ± 0.54 bc	0.68 ± 0.01 cd	0.92 ± 0.03ab	5.20 ± 0.47 abc	4.79 ± 0.39 ab
		10	4.57 ± 0.00 fg	0.70 ± 0.02 bc	0.92 ± 0.03 ab	4.98 ± 0.34 bcd	4.57 ± 0.37 abc
		15	6.16 ± 1.00 de	0.71 ± 0.02 bc	0.92 ± 0.02 ab	4.35 ± 0.56 de	4.02 ± 0.30 cd
720	1F	0	3.55 ± 0.63 g	0.65 ± 0.04 e	0.82 ± 0.03 c	2.24 ± 0.47 g	1.83 ± 0.08 g
		5	4.60 ± 0.90 fg	0.62 ± 0.02 f	0.82 ± 0.05 c	2.82 ± 0.40 fg	2.27 ± 0.07 fg
		10	6.32 ± 0.30 cde	0.58 ± 0.03 g	0.81 ± 0.05 c	3.63 ± 0.33 ef	2.93 ± 0.14 ef
		15	6.87 ± 0.59 bcd	0.54 ± 0.04 h	0.77 ± 0.03 d	3.64 ± 0.37 ef	2.78 ± 0.17 ef
	2F	0	10.29 ± 0.19 a	0.57 ± 0.03 g	0.73 ± 0.06 e	5.88 ± 0.64 a	4.30 ± 0.33 bc
		5	10.83 ± 0.28 a	0.54 ± 0.03 h	0.72 ± 0.04 e	5.80 ± 0.45 ab	4.22 ± 0.33 bc
		10	9.78 ± 0.94 a	0.49 ± 0.02 i	0.67 ± 0.04 f	4.77 ± 0.43 cd	3.19 ± 0.26 e
		15	8.08 ± 0.87 b	0.53 ± 0.03 h	0.67 ± 0.07 f	4.27 ± 0.43 de	2.89 ± 0.35 ef
580			5.88 ± 1.58 b	0.71 ± 0.03 a	0.92 ± 0.02 a	4.34 ± 0.50 a	4.00 ± 0.44 a
720			7.54 ± 1.92 a	0.56 ± 0.06 b	0.75 ± 0.08 b	4.13 ± 0.45 a	3.05 ± 0.52 b
1F			5.27 ± 1.79 b	0.66 ± 0.08 a	0.86 ± 0.07 a	3.40 ± 0.49 b	2.92 ± 0.48 b
2F			8.15 ± 1.24 a	0.61 ± 0.09 b	0.81 ± 0.12 b	5.08 ± 0.44 a	4.14 ± 0.44 a
0			6.83 ± 1.79 a	0.64 ± 0.05 a	0.85 ± 0.09 a	4.31 ± 0.58 ab	3.67 ± 0.36 a
5			7.06 ± 1.84 a	0.64 ± 0.08 a	0.84 ± 0.09 ab	4.40 ± 0.45 a	3.69 ± 0.40 a
10			6.43 ± 1.34 a	0.63 ± 0.11 a	0.83 ± 0.11 bc	4.30 ± 0.44 ab	3.53 ± 0.48 ab
15			6.51 ± 1.19 a	0.63 ± 0.10 a	0.82 ± 0.11 c	3.96 ± 0.46 b	3.23 ± 0.43 b

Values are expressed as the mean (n = 2) ± standard deviation. Mean values bearing different letters in the same row denote statistical differences (a > b > c …, etc.). PF—psyllium fiber, 1F—one-phase baking method, 2F—two-phase baking method.

## Data Availability

Results will be available from the corresponding author.

## Supplementary Materials

The following supporting information can be downloaded at https://www.mdpi.com/article/10.3390/foods12193534/s1, Figure S1: Photo of the crumb of bread baked with the single-phase (1F) and two-phase (2F) method with RF type 580 and 720 with different level of PF (0%, 5%, 10%, 15%); Table S1: Significant Pearson’s correlation coefficients (significance level α ≤ 0.05).
