# Supplementary material for: Mixed Psyllium Fiber Improves the Quality, Nutritional Value, Polyphenols and Antioxidant Activity of Rye Bread
Source: Foods. 2023 Sep 22;12(19):3534. doi: 10.3390/foods12193534 (PMC10572817; doi:10.3390/foods12193534)
Supplement: Supplementary file 1 [file foods-12-03534-s001.zip › Fig S1. Breads.pdf]

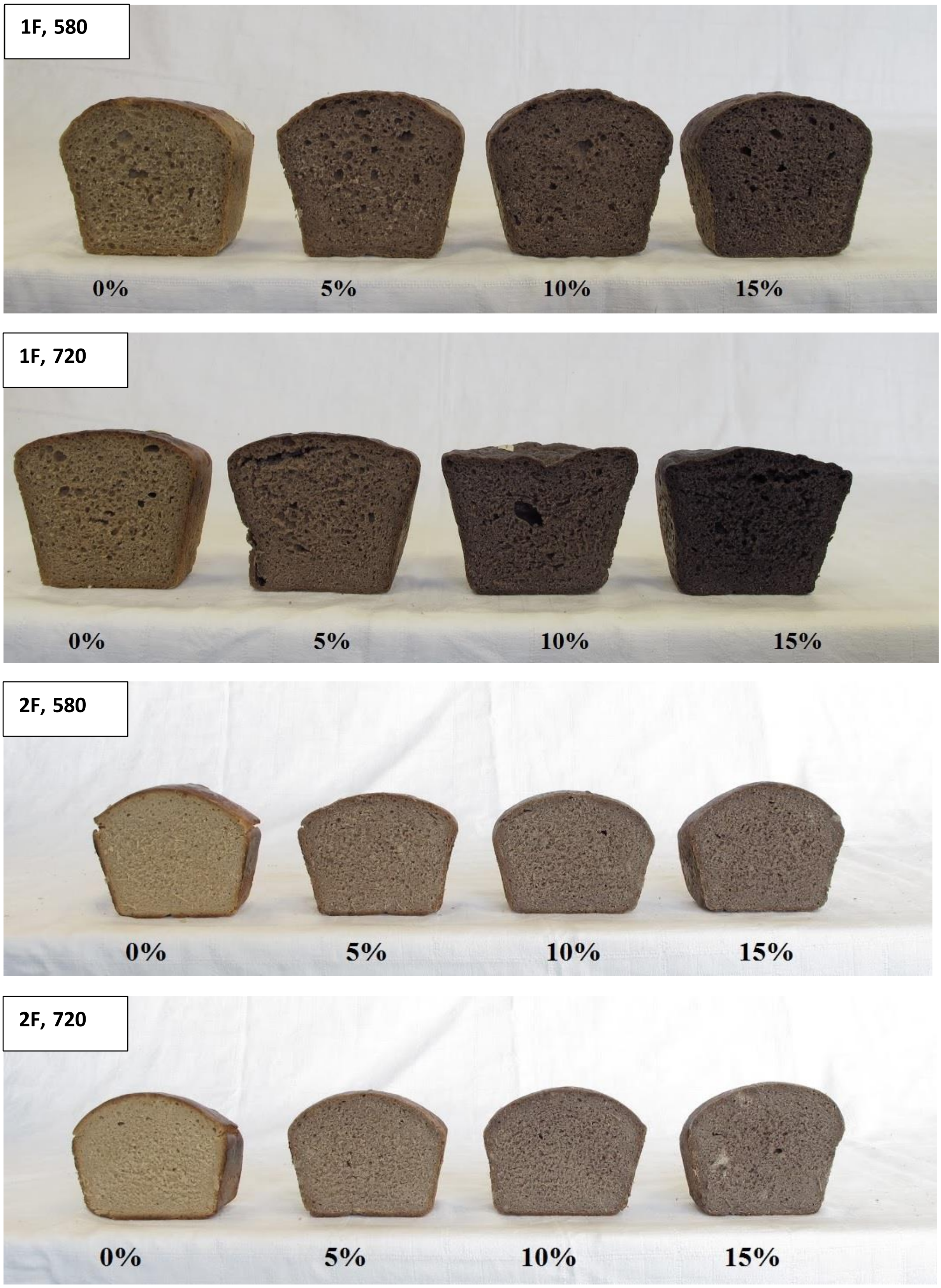

Figure S1. Photo of the crumb of bread baked with the single-phase (1F) and two-phase (2F) method with RF type 580 and 720 with different level of PF (0%, 5%, 10%, 15%).
