# Supplementary material for: Mixed Psyllium Fiber Improves the Quality, Nutritional Value, Polyphenols and Antioxidant Activity of Rye Bread
Source: Foods. 2023 Sep 22;12(19):3534. doi: 10.3390/foods12193534 (PMC10572817; doi:10.3390/foods12193534)
Supplement: Supplementary file 1 [file foods-12-03534-s001.zip › Table S1.pdf]

Table S1. Significant Pearson's correlation coefficients (significance level $\alpha \leq 0{,}05$)

	Ash	TDF	Hardness	Cohesiveness	Springiness	Gumminess	Chewiness	L* crust	a* crust	b* crust	L* crumb	a* crumb	b* crumb	overbake	specific volume	crumb porosity	total protein	ABTS	FRAP	TPC
Ash		0,980	0,053	-0,446	-0,505	-0,218	-0,442	-0,716	-0,902	-0,836	-0,859	0,520	-0,850	0,535	-0,316	0,331	0,689	0,827	0,848	0,351
TDF	0,980		0,020	-0,488	-0,546	-0,296	-0,536	-0,752	-0,886	-0,840	-0,868	0,521	-0,828	0,574	-0,288	0,335	0,730	0,823	0,900	0,246
Hardness	0,053	0,020		-0,669	-0,619	0,799	0,440	0,146	0,149	-0,121	0,132	-0,281	0,190	-0,346	-0,729	0,610	0,308	0,199	0,228	0,041
Cohesiveness	-0,446	-0,488	-0,669		0,936	-0,204	0,262	0,387	0,337	0,573	0,241	0,062	0,066	-0,038	0,488	-0,505	-0,837	-0,300	-0,668	0,267
Springiness	-0,505	-0,546	-0,619	0,936		-0,144	0,362	0,455	0,376	0,665	0,345	-0,095	0,075	-0,120	0,531	-0,671	-0,874	-0,376	-0,757	0,238
Gumminess	-0,218	-0,296	0,799	-0,204	-0,144		0,868	0,524	0,424	0,251	0,333	-0,301	0,248	-0,595	-0,648	0,407	-0,178	0,044	-0,218	0,409
Chewiness	-0,442	-0,536	0,440	0,262	0,362	0,868		0,732	0,579	0,572	0,493	-0,349	0,257	-0,638	-0,341	0,026	-0,595	-0,146	-0,580	0,517
L* crust	-0,716	-0,752	0,146	0,387	0,455	0,524	0,732		0,868	0,884	0,669	-0,357	0,523	-0,689	-0,121	-0,210	-0,748	-0,511	-0,752	0,137
a* crust	-0,902	-0,886	0,149	0,337	0,376	0,424	0,579	0,868		0,874	0,814	-0,526	0,800	-0,612	-0,032	-0,081	-0,701	-0,649	-0,734	-0,189
b* crust	-0,836	-0,840	-0,121	0,573	0,665	0,251	0,572	0,884	0,874		0,736	-0,443	0,568	-0,434	0,146	-0,375	-0,858	-0,626	-0,799	-0,092
L* crumb	-0,859	-0,868	0,132	0,241	0,345	0,333	0,493	0,669	0,814	0,736		-0,834	0,881	-0,611	0,253	-0,223	-0,494	-0,769	-0,729	-0,329
a* crumb	**0,520**	**0,521**	-0,281	0,062	-0,095	-0,301	-0,349	-0,357	-0,526	-0,443	-0,834		-0,656	0,319	-0,049	0,042	0,204	0,477	0,343	0,314
b* crumb	-0,850	-0,828	0,190	0,066	0,075	0,248	0,257	0,523	0,800	0,568	0,881	-0,656		-0,552	0,185	0,005	-0,292	-0,763	-0,572	-0,555
Overbake	0,535	0,574	-0,346	-0,038	-0,120	-0,595	-0,638	-0,689	-0,612	-0,434	-0,611	0,319	-0,552		-0,004	0,115	0,298	0,411	0,613	-0,135
Specific volume	-0,316	-0,288	-0,729	0,488	0,531	-0,648	-0,341	-0,121	-0,032	0,146	0,253	-0,049	0,185	-0,004		-0,754	-0,174	-0,501	-0,430	-0,373
Crumb porosity	0,331	0,335	0,610	-0,505	-0,671	0,407	0,026	-0,210	-0,081	-0,375	-0,223	0,042	0,005	0,115	-0,754		0,426	0,476	0,576	0,104
Total protein	0,689	0,730	0,308	-0,837	-0,874	-0,178	-0,595	-0,748	-0,701	-0,858	-0,494	0,204	-0,292	0,298	-0,174	0,426		0,451	0,822	-0,245
ABTS	0,827	0,823	0,199	-0,300	-0,376	0,044	-0,146	-0,511	-0,649	-0,626	-0,769	0,477	-0,763	0,411	-0,501	0,476	0,451		0,770	0,452
FRAP	0,848	0,900	0,228	-0,668	-0,757	-0,218	-0,580	-0,752	-0,734	-0,799	-0,729	0,343	-0,572	0,613	-0,430	0,576	0,822	0,770		-0,037
TPC	0,351	0,246	0,041	0,267	0,238	0,409	0,517	0,137	-0,189	-0,092	-0,329	0,314	-0,555	-0,135	-0,373	0,104	-0,245	0,452	-0,037	
